# The Order of Cancer: A Theory of Malignant Progression by Inverse Morphogenesis

**DOI:** 10.3389/fonc.2019.00416

**Published:** 2019-05-29

**Authors:** Michael Höckel, Ulrich Behn

**Affiliations:** ^1^Department of Gynecology, Women's and Children's Center, University of Leipzig, Leipzig, Germany; ^2^Leipzig School of Radical Pelvic Surgery, University of Leipzig, Leipzig, Germany; ^3^Department of Gynecology and Obstetrics, University of Essen, Essen, Germany; ^4^Faculty of Physics and Earth Sciences, Institute of Theoretical Physics, University of Leipzig, Leipzig, Germany

**Keywords:** cancer, cancer field, surgical oncology, malignant progression, morphogenesis, complex systems, self-organization

## Abstract

Local spread patterns of malignant tumors follow permissive tissue territories, i.e., cancer fields, as shown for cervical and vulvar carcinoma. The cancer fields are associated in reverse order to the mature derivatives of the morphogenetic fields instrumental in the stepwise development of the tissue from which the tumor arose. This suggests that cancer progression may be linked to morphogenesis by inversion of the cellular bauplan sequence. Successive attractor transitions caused by proliferation-associated constraints of topobiological information processing are proposed for both morphogenesis and cancer. In morphogenesis these transitions sequentially activate bauplans with increasing complexity at decreasing plasticity restricting the permissive territories of the progenitor cell populations. Somatic mutations leading to cell proliferation in domains normally reserved for differentiation trigger the inverse cascade of bauplan changes that increase topobiological plasticity at decreased complexity and stepwise enlarge the permissive territory of neoplastic cells consistent with the clinical manifestations of cancer. The order provided by the sequence of attractor transitions and the defined topography of the permissive territories can be exploited for more accurate tumor staging and for locoregional tumor treatment either by surgery or radiotherapy with higher curative potential.

## Introduction

Most cancer researchers accept a model of oncogenesis that is based on the principle of random variation and selection. Following a neo-Darwinian concept random genomic mutations selected for reproductive fitness within the ecosystem of the cell's microenvironment transform normal cells into cancer cells. Sequential additional mutations drive their malignant progression by clonal expansion to the invasive and metastatic phenotypes (1). A stochastic process is also assumed for the local spread of a malignant tumor rendering the paths of cancer cells that leave the tumor's core region unpredictable and therefore isotropic. Consequently, oncologists consider the resection or irradiation of the macroscopic tumor with a metrically defined circumferential margin of cancer-free tissue necessary for local control (2).

However, a variety of experimental and clinical facts are not consistent with these tenets. Although distinct genomic mutations providing growth advantage by gain of function in oncogenes and loss of function in tumor suppressor genes have been identified for early steps of oncogenesis, additional somatic mutations conclusively associated with and specific for the metastatic process could not be demonstrated so far (3, 4). Normalization of invasive and metastatic tumors to mature differentiated tissues through interaction with embryonic microenvironments (5–7) and *in vitro* (8) as well as the neoplastic transformation of normal cells and, conversely, the regression of oncogene-induced tumors by modulation of transmembrane potential (9, 10) cannot be explained by a neo-Darwinian evolution model without *ad-hoc* amendments. We have presented evidence from the pattern analysis of carcinoma of the uterine cervix and the vulva that the local spread of these tumors is not compatible with a random model (11, 12). If this model was valid, the resection of a local tumor with a wider margin of healthy tissue should achieve a higher local tumor control than resection with closer margins. Yet the clinical results of thousands of cancer courses show that this is not the case [for review see (13)]. Likewise, the establishment of a local staging system for the different tumor entities contradicts the concept of isotropic cancer propagation (14).

## The Theory

We propose a theory of cancer that involves a principle of order established through the self-organizing process of morphogenesis. Whereas clinical cancer initiation is caused by somatic mutations, malignant progression is considered to result from attractor-driven “inverse morphogenesis,” an epigenetic process possibly modulated by but not dependent on additional mutations. In the most general terms the theory is based on the following assumptions: Immature metazoic cells are self-organizing complex systems assembling collectives (tissues), functional multipopulation units (organs) and, finally, organisms. All stable states, from the levels of living cell to living organism represent attractors in the corresponding state spaces of the cell's genetic regulatory network established by evolution (15, 16). Instrumental for this complex regulatory system is both, the genome and its three-dimensional epigenomic organization by hierarchical folding (17). Morphogenesis is considered as a process determined by “*forward” genomic folding* increasing the complexity of topobiological information processing at decreased plasticity along with the emergence of higher-level attractors such as tissue, organ, and organism attractors. Cancer is proposed to involve the reverse process of “*backward” genomic unfolding* decreasing topobiological complexity at increasing plasticity with the dominance of the cancer cell attractors over the higher-level attractors. Both forward and backward transitions in chromatin organization are induced by proliferation-driven attractor destabilization.

### Morphogenesis by Proliferation-Driven Genomic “Forward” Folding

We assume that morphogenesis is executed by cell populations with a hierarchical sequence of a common *bauplan*, a layer of the cell's genetic regulatory network as self-organizing complex system stabilized by dynamical attractors. The German term bauplan was introduced into the field of embryology initially to describe archetypical body plans of different species (18). The bauplan as used here in a more specific sense enables the cells to perceive *topobiological information* (19) presented by the surrounding *morphogenetic field* through signaling (e.g., epitopes, chemical gradients) and physical phenomena (e.g., pressure, tension, bioelectrical events) and to respond with programmed activities such as proliferation, migration, aggregation, differentiation, quiescence, apoptosis, etc. to assemble tissue structures. Simultaneously, the cells produce topobiological information by themselves generating collectively the morphogenetic field.

Besides the bauplan, the cell's genetic regulatory network determines at different other layers biological features such as metabolism and energy production which are also relevant for morphogenesis. All layers interact with each other but only the bauplan is considered here. Each bauplan can be characterized by two features: (i) complexity of topobiological information that is processed and (ii) plasticity enabling cells to adapt their bauplans to the topobiological information provided by the morphogenetic fields of abutting cell populations. Bauplan changes due to chromatin reorganization that enhance the complexity of topobiological information processing simultaneously decrease its plasticity or adaptability. A totipotent early blastomere cell at the lowest level of morphogenetic hierarchy is characterized by maximum bauplan plasticity, i.e., all potential bauplans inherent in the cell's genome can be realized by interaction with the corresponding microenvironments. Yet, the complexity of topobiological information processing of the totipotent cell is minimal involving mainly physical interactions (18). The adult cells as the highest level of the morphogenetic hierarchy exhibit minimal or no bauplan plasticity any longer but execute the maximum complexity of topobiological information processing necessary to maintain tissue homeostasis (20). The final bauplan has to fine-tune the substitution for continuous cell loss and in concert with the innate and adaptive immune systems to preserve tissue integrity despite assaults by infectious agents, injury, and host-generated threats.

During morphogenesis the proliferation of the cells increases the topobiological information to be processed by enhancing signals through an increase of cellular surfaces, extracellular matrix and gradients of soluble molecules as well as mechano-transduction that force the cells to respond and thus provides progressive constraints to the system driving its attractor toward instability. At the point of instability bifurcation into new attractors with higher complexity of information processing at decreased plasticity occurs (21, 22). Each attractor translates into a new cell type with a new bauplan that responds to the topobiological information of its associated, spatially more limited morphogenetic field with higher capacity achieving a temporary stabilization of the system.

**Figure 1:**
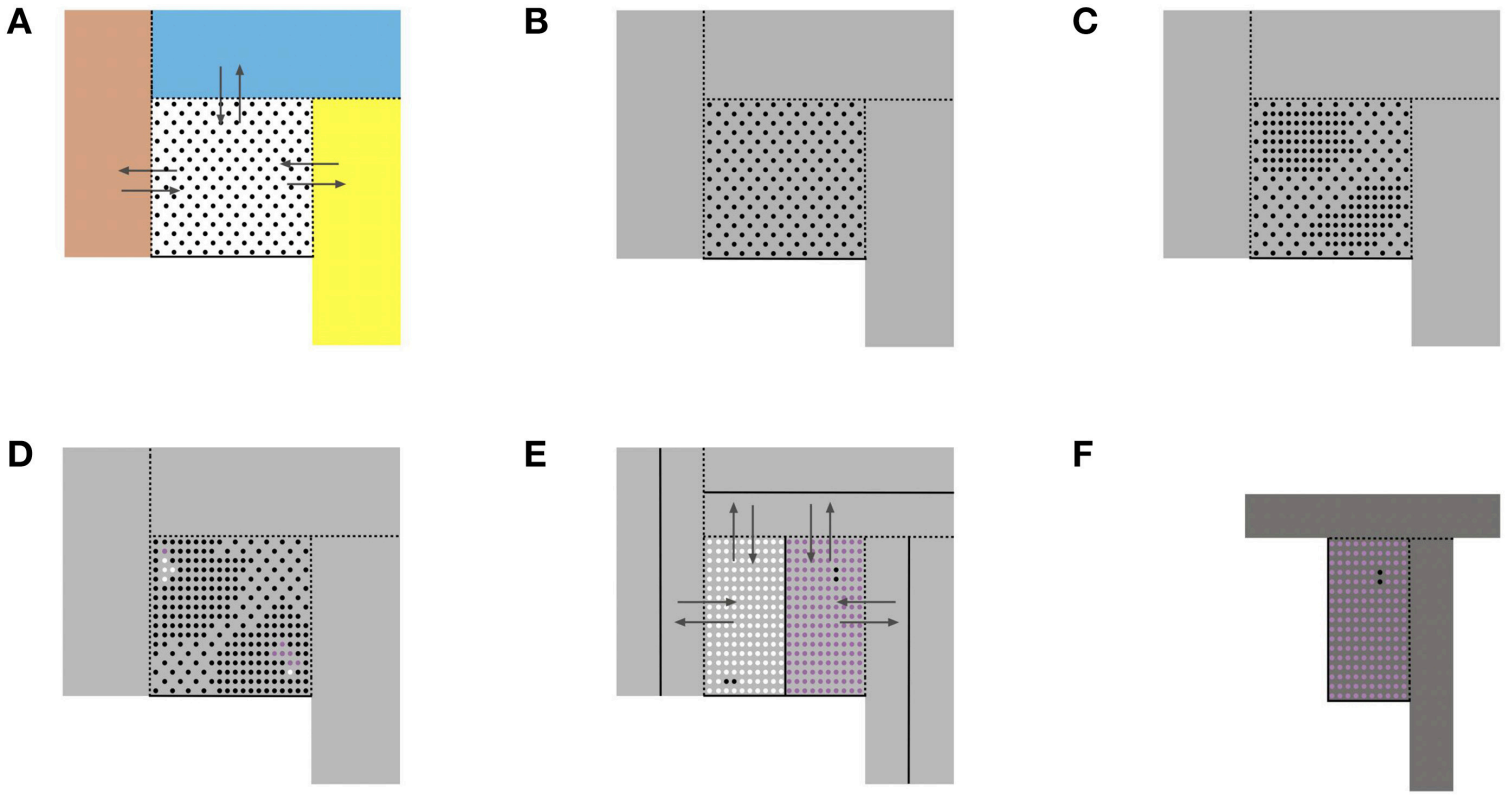
Schematic representation of some basic aspects of morphogenesis as self-organizing process. **(A)** A reference cell population (dots) with a common bauplan executed in a morphogenetic field (white square) abuts to other cell populations occupying their morphogenetic fields (colored rectangles). The reference cells have the plasticity to adapt to the bauplan of the adjacent cell populations and to integrate into the adjacent morphogenetic fields. Likewise, abutting cells may enter the reference cell's morphogenetic field changing their bauplans correspondingly (arrows). Dashed lines indicate transgressable borders, solid lines cannot be transgressed. **(B)** As a consequence, the morphogenetic metafield of the reference cell population is set up by its own field and those of all abutting populations that mutually exchange cells (gray area). **(C)** Cell proliferation increases the constraints due to the gain of topobiological information to be processed and destabilizes the epigenomic state. **(D)** Bifurcation into two new epigenomic attractors leads to two daughter cell populations (white and purple dots) with bauplans of higher complexity of information processing but lower plasticity. Likewise, cell populations abutting the reference population undergo proliferation-induced bifurcations into two daughter populations with different bauplans (not shown). **(E)** The new daughter populations with lower plasticity segregate from each other and form non-transgressable lineage boundaries between them (solid lines). Therefore, the number of transgressable boundaries (dashed lines) decreases. **(F)** The morphogenetic metafield for the purple population is indicated as darker gray area. 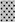 Reference cell population X; 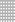 Daughter cell population (X+1)_1_; 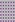 Daughter cell population (X+1)_2_; 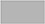 Morphogenetic metafield X; 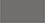 Morphogenetic metafield (X+1)_2_.

Due to the plasticity of chromatin organization that enables embryonic cells to change their bauplan when facing the morphogenetic fields of the abutting populations, the interactive domain of an embryonic cell type includes the morphogenetic field of its own population and those of the abutting cell populations. This extended territory is termed here *morphogenetic metafield*. Figure 1 schematically illustrates a morphogenetic step of a cell population exchanging cells with abutting populations. The bifurcation process is reiterated by continuous cell proliferation until the maximum complexity at minimum plasticity of the system is reached with the mature tissue state in homeostasis. Based on morphological features in the initial steps of human morphogenesis (23) and in the complete development of the female genital tract (23–28) we postulate that during the embryonic period (Carnegie stages 1–23) each bifurcation results in two new attractor states producing two daughter cell types with different non-interchangeable bauplans. Their populations are consequently separated by a non-transgressable lineage border. At the end of the embryonic period the plasticity of bauplan adaptation between abutting cell populations is exhausted. During the fetal period continued morphogenesis occurs exclusively within the cell lineage's own morphogenetic field by specification along defined axes and allometric growth forming multiple subcompartments at different levels.

**Figure 2:**
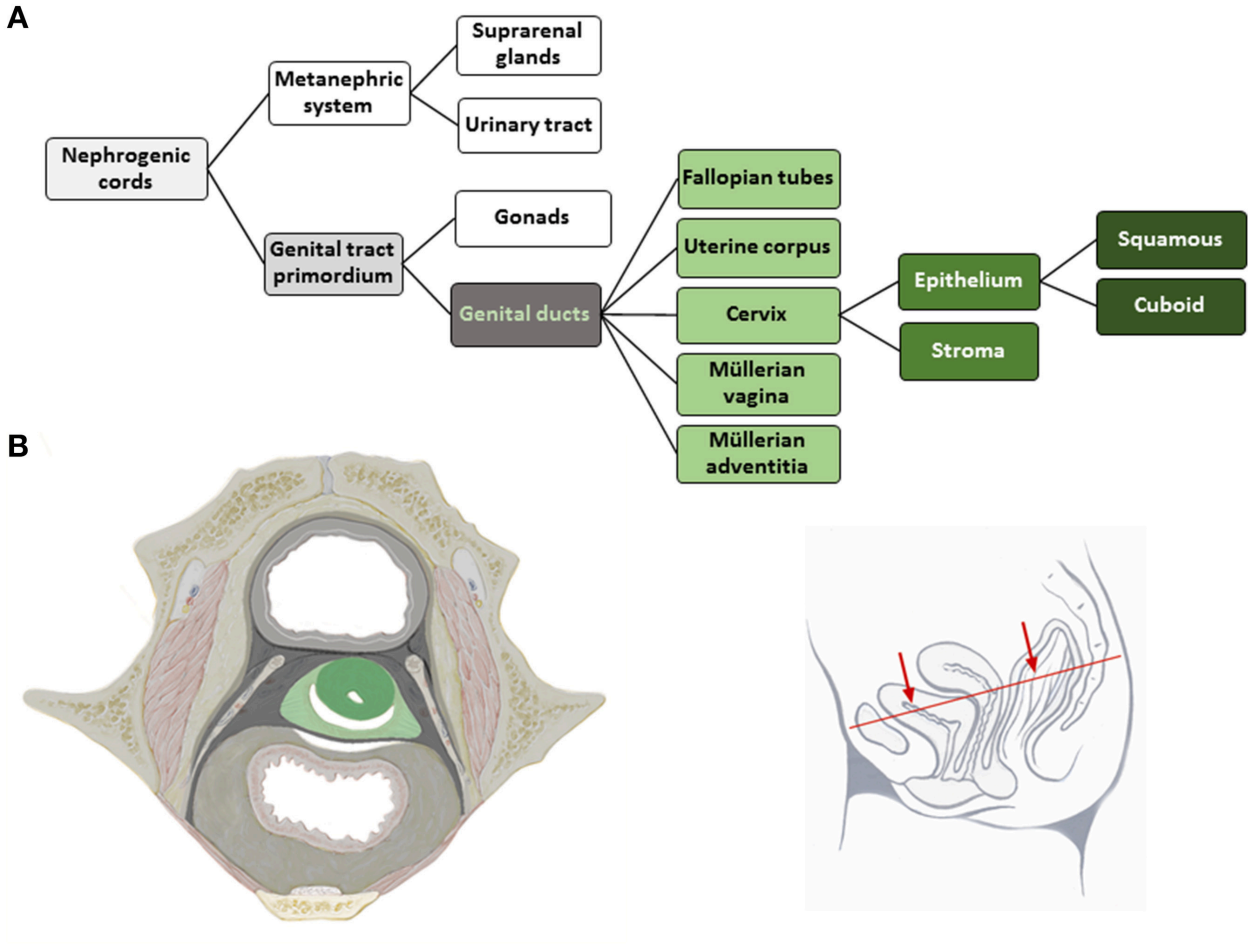
**(A)** Terminal steps of the ontogenetic tree for the development of the human uterine cervix from the nephrogenic cords. During the developmental stages highlighted in shades of gray epigenomic plasticity allows bauplan adaptations of abutting cell populations. Light green lettering indicates the cell populations of a morphogenetic compartment at the end of the embryonic period, darker green coloring indicates subcompartments. The cervical epithelium subcompartment segregates further into squamous and cuboid types. **(B)** Ontogenetic anatomical map at the transverse section through the female midpelvis as shown with the inset. Color-coding indicates the mature tissue derivatives of the terminal developmental steps. Dark green, macroscopic subcompartment; light green, compartment; dark, middle and light gray, sequential morphogenetic metafields. Morphological details of the developmental path from the nephrogenic cords to the cervical epithelia are given in the Supplement [**(B)** adapted from (6), copyright permission granted by Elsevier].

**Figure 3:**
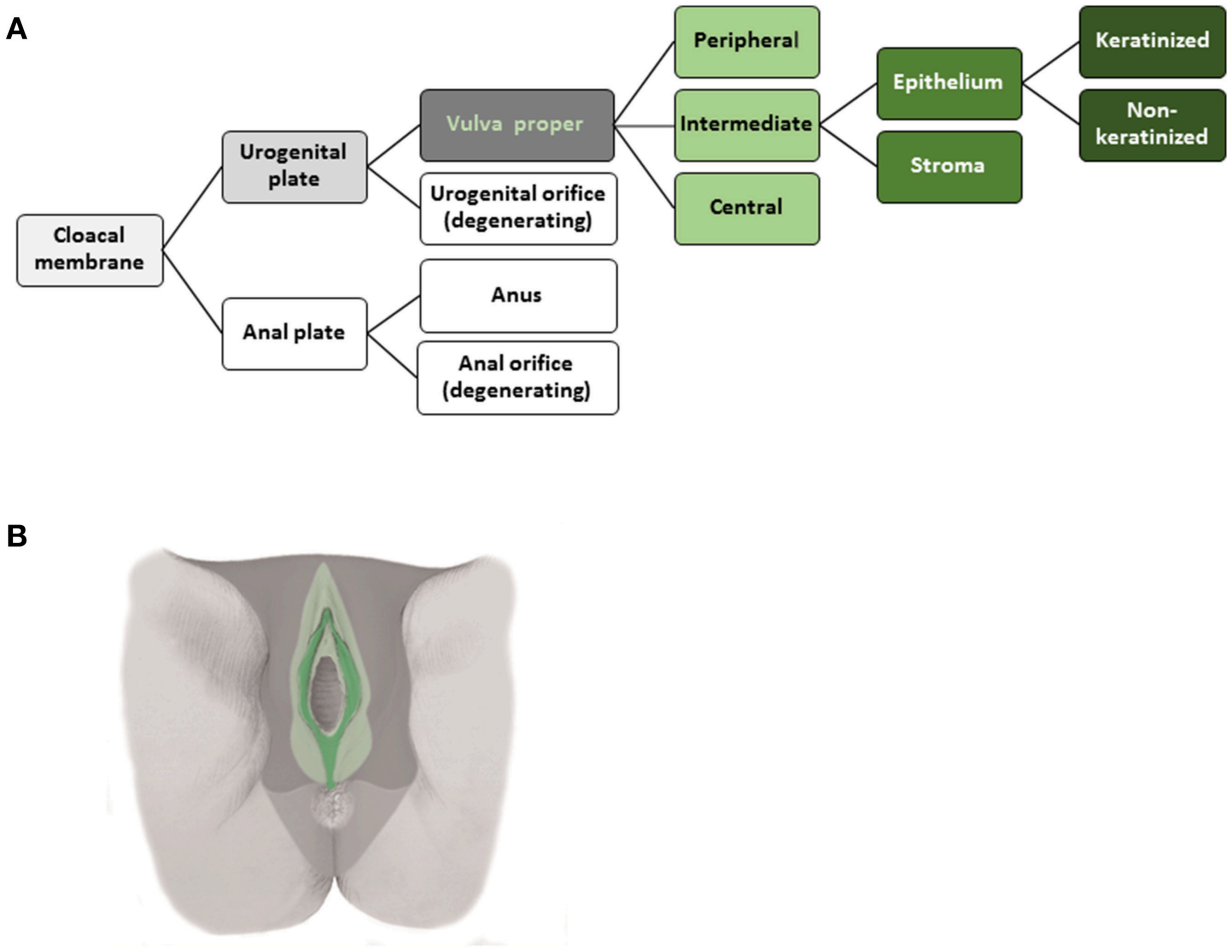
**(A)** Terminal steps of the ontogenetic tree for the development of the intermediate subcompartment of the vulva from the cloacal membrane endoderm. During the developmental stages highlighted in shades of gray epigenomic plasticity allows bauplan adaptations of abutting cell populations. Light green lettering indicates the cell populations of a morphogenetic compartment at the end of the embryonic period, darker green coloring indicates subcompartments. The epithelium of the intermediate vulvar subcompartment segregates further into keratinized and non-keratinized types. **(B)** Ontogenetic anatomical map of the intermediate subcompartment of the vulva shown at the perineal surface. Color-coding indicates the mature tissue derivatives of the terminal developmental steps. Dark green, macroscopic subcompartment; light green, compartment; dark, middle and light gray, sequential morphogenetic metafields. Morphological details of the developmental path from the cloacal membrane endoderm to the epithelia of the middle vulvar subcompartment are given in the Supplement [**(B)** adapted from (7), copyright permission granted by Elsevier].

The tissue structures established within the morphogenetic field of a reference cell population during development can be followed morphologically and placed hierarchically into an *ontogenetic tree*. The ontogenetic trees for the human cervix uteri (as subcompartment of the Müllerian system) and of the vulva from the phylotypic stage to maturity are shown in Figures 2A, 3A. The mature tissue derivatives of the corresponding morphogenetic metafields, compartments, and subcompartments are color-coded to create *ontogenetic anatomic maps*. Figure 2B illustrates the ontogenetic anatomical map of the uterine cervix in a midpelvic axial plane as indicated. The ontogenetic anatomical map of the middle subcompartment of the vulva at the perineal surface is shown in Figure 3B. Morphological details of the developmental paths in the morphogenesis of the uterine cervix and vulva are given in the Supplement.

### Cancer Progression by Proliferation-Driven Genomic “Backward” Unfolding

We further postulate that under clinically relevant conditions a malignant tumor is initiated by (epi-)genetic alterations increasing overall cell proliferation and perturbing the terminal bauplan of the affected clonogenic cell enabling cell divisions at domains normally restricted to differentiation. For epithelial cancers this manifestation is microscopically observable in dysplasia/carcinoma *in situ*. Distinct genetic alterations leading to a gain of function of oncogenes and loss of function of tumor suppressor genes are known for long as so called “driver” mutations (29). Continued proliferation at non-permissive sites surpasses the cell's capacity of topobiological information processing for that level of complexity and destabilizes its terminal attractor. Transition into the penultimate attractor achieves temporary stabilization of the genetic regulatory network as complexity decreases and therefore less topobiological information has to be processed. However, the bauplan of that state expands the permissive domain of the proliferating cancer cells, i.e., the cancer field is widened to the corresponding morphogenetic territory. Persistent proliferation of the cancer cells within their enlarged permissive territory of mature non-cancerous tissue drives the genetic regulatory network again to its limits of topobiological information processing and destabilizes the penultimate attractor. Contrary to the situation during development the microenvironment of the mature organism does no longer allow “forward” genomic folding of the cancer cell to reach higher order attractors (although this may be induced experimentally). The backtrack in the attractor landscape of the cancer cell populations that increase topobiological plasticity is self-propelling and leads to the loss of stability of the tissue, organ, and finally organism attractors causing the death of the individual. Once cancer is initiated by somatic mutations causing uncontrolled proliferation and bauplan alterations further mutations are no longer necessary to maintain malignant progression.

**Figure 4:**
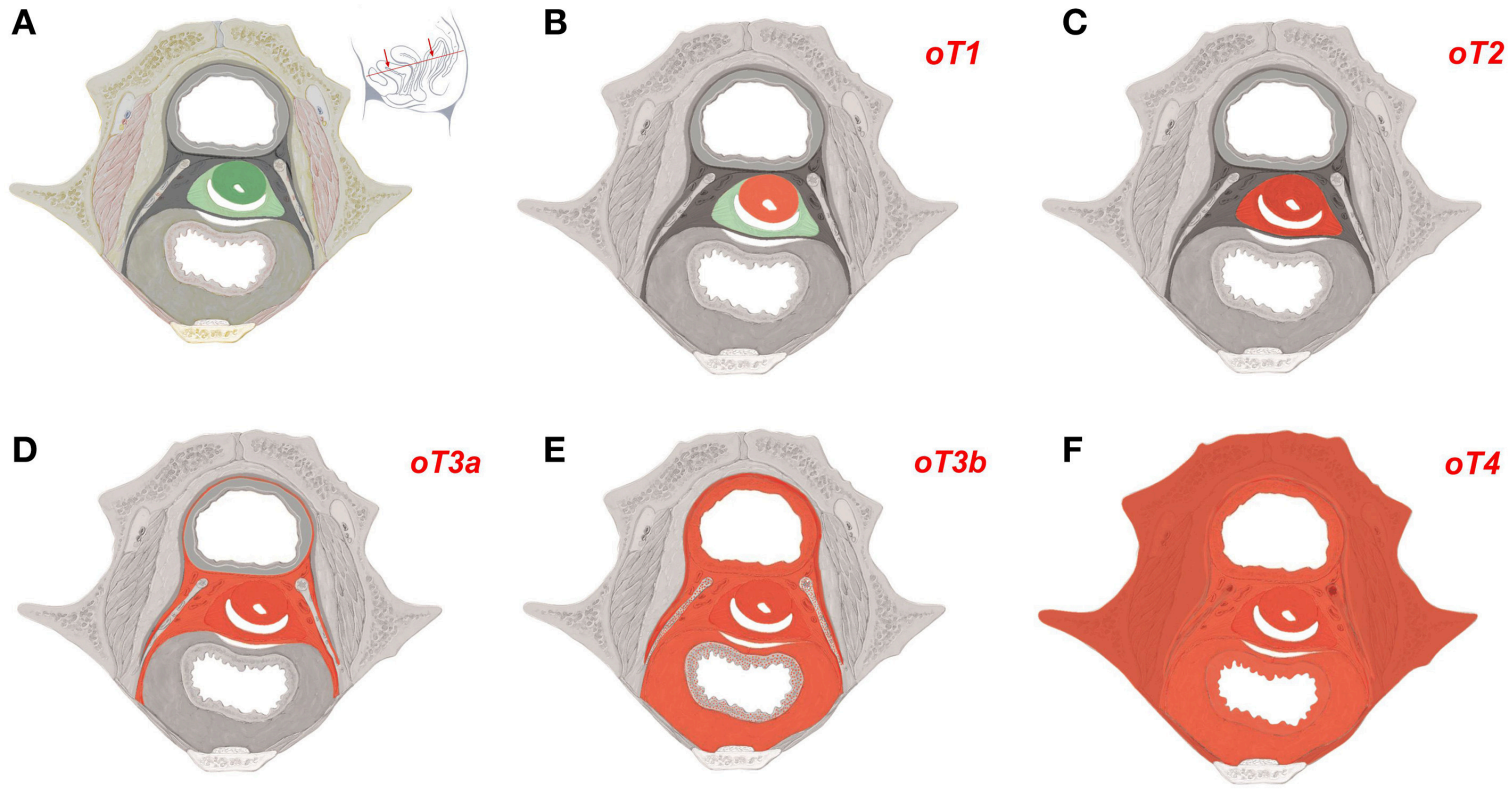
Ontogenetic determination of local tumor spread in cervical carcinoma. **(A)** Ontogenetic anatomy of the cervix demonstrated by a transverse section of the female ischiopubic endopelvis specified with the inset. Color-coding denotes the mature tissue derivatives of the last five macro-developmental steps. Dark green, macroscopic subcompartment; light green, compartment; dark gray, compartment field; middle gray, late metafield; light gray, early metafield. **(B–F)** Ontogenetic cancer fields for carcinoma of the cervix stages oT1-oT4. Cancer fields are colored red. Other colors correspond to **(A)**. Up to ontogenetic stage 3a the permissive cancer field corresponds completely to the mature tissues derived from the corresponding morphogenetic fields. From oT3b onward the cancer fields exceed the tissue derivatives of the morphogenetic metafields as cells from abutting populations having entered the reference cell population during the earlier steps of morphogenesis regain their bauplan plasticity by the epigenomic unfolding. These cancer cells are permissive in the tissue derivatives of the morphogenetic fields of the abutting cell populations in addition to those of the reference population (dotted areas). At ontogenetic stage 4 almost all locoregional tissues can be infiltrated by the cancer cells. [Figure adapted from (6), copyright permission granted by Elsevier].

**Figure 5:**
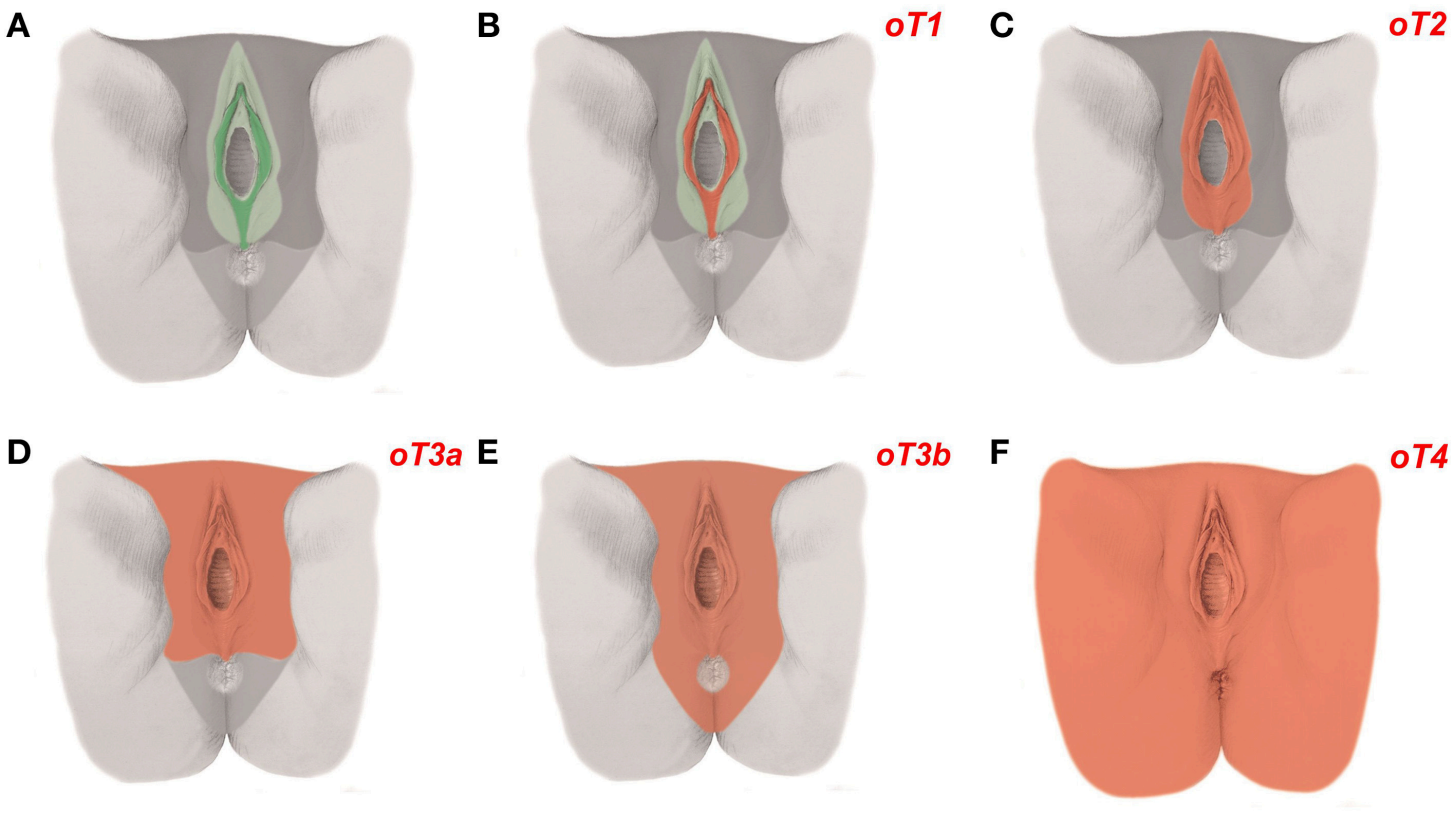
Ontogenetic determination of local tumor spread in carcinoma of the vulva. **(A)** The ontogenetic anatomy of the vulva with its intermediate subcompartment shown at the perineal surface. Color-coding denotes the mature tissue derivatives of the last five macro-developmental steps. Dark green, macroscopic subcompartment; light green, compartment; dark gray, compartment field; middle gray, late metafield; light gray, early metafield. **(B–F)** Ontogenetic cancer fields for vulvar carcinoma, stages oT1-4. Cancer fields are colored red. Other colors correspond to **(A)**. Each vulvar subcompartment (peripheral, intermediate, and central) defines its oT1 cancer field. Only the cancer field of the intermediate subcompartment is shown [Figure adapted from (7), copyright permission granted by Elsevier].

As the mature tissue derivatives of the hierarchical morphogenetic (meta-)fields can be determined and expressed in ontogenetic anatomical maps for each tissue of interest, the cancer fields obeying to the reverse hierarchy are morphologically distinct and represent an element of order in the malignant process. The cancer fields for carcinoma of the cervix and the vulva (middle subcompartment) are demonstrated with Figures 4, 5.

Associations between morphogenesis and cancer spread have also been demonstrated for malignancies such as carcinoma of the rectum (30), pancreas (31), face (32) as well as for gliomas (33). Topobiological information shared by the lymph node and its peripheral target tissue is considered as locoregional link between a cancer field and its surveilling lymph node regions. Malignant progression beyond the phylotypic stage of inverse morphogenesis makes the majority of distant tissues permissive for metastatic dissemination and colonization.

## Discussion

The association between development and malignant disease has been proposed for long dating back to the beginnings of cellular pathology in the nineteenth century and has given rise to many embryology-based cancer theories (34–37). Modeling morphogenesis and cancer as complex systems of genetic regulatory networks stabilized by attractors has been elaborated by Kauffman (15). Huang and Ingber (16) assume that cancer cells represent genetic regulating networks trapped in embryonic state attractors. The association between morphogenesis and the dynamical patterns of local spread of carcinomas of the uterine cervix and vulva lead us to hypothesize a bifurcational hierarchy of attractor transitions occurring as “forward” genomic folding with morphogenesis and as “backward” unfolding of the altered epigenome in malignant progression. The forward process increases the complexity of topobiological information processing at decreased plasticity to adapt to a different microenvironment and limits the permissive territories of cell populations. The backward process is characterized by the opposite events. Proliferation-associated reinforced topobiological information processing is proposed to induce the attractor transitions in both situations.

The theory is consistent with the many known embryonic pathways active in cancer (38, 39). Malignant phenotype normalization may result from “forward” folding of the genome of the cancer cell forced by distinct microenvironments *in vivo* or *in vitro* (5–8, 10). The proposed self-perpetuating process of cancer progression explains its independence from genetic mutations beyond those necessary for cancer initiation and the fact that those mutations predicted from the neo-Darwinian model have not been detected. However, additional “driver” mutations may modulate the dynamics of the process of epigenetic backtracking, increase heterogeneity and are therefore clinically relevant. The anisotropic tumor permeation and the missing significance of surgical margin width to predict local tumor recurrence can be understood from the allocation of topographically defined permissive tissue territories (cancer fields) to cancer cells according to their state of malignant progression. Surgical margin width is only predictive for tumor relapse within an individual cancer field. A close margin at the border of the cancer field is prognostically irrelevant. As the relation of the minimal margin to the extension of the cancer field is not known to the conventional surgeon or to the pathologist, its predictive importance is subject to chance and therefore not robust (13).

Allocating the local spread of a malignant tumor to the topography of the mature tissues derived from the morphogenetic fields of the stepwise development of the tissue from which the cancer originated allows the *ontogenetic staging* of the individual cancer and its precise surgical resection or radiation respecting its cancer field. Ontogenetic staging has been shown to be superior to current empirical staging to predict the outcome of cancers diagnosed with a broad spectrum of disease courses such as cervix and vulva carcinoma (11, 12). *Cancer field resection* preserves functionally or esthetically important tissues even in immediate vicinity to the macroscopic tumor if they do not belong to the cancer field. Dispensing with adjuvant radiation is justified if cancer field resection has been adequately performed. Thus, clinical translation of the theory offers a significant potential to improve the outcome of cancer treatment in terms of locoregional tumor control and treatment-related morbidity as already demonstrated for carcinoma of the rectum (40), uterine cervix (41), and vulva (12).

## Perspective

The most stringent test of the theory would be the demonstration of common interphase genome folding motifs both in ontogenetically defined normal (stem) cells and in cancer cells of the corresponding ontogenetic stage. Although methods to study genome organization with high resolution (i.e., 100 kb scale) have been developed (42) this approach appears beyond present feasibility. However, gene expression analyses of cancer cells of distinct ontogenetic stages to be compared with those of the normal cells of the corresponding state in morphogenesis appear realizable with the methods currently available (43). Likewise, the identification of common molecular epitopes, e.g., chemokines, integrins, semaphorins and plexins in mature cells derived from a distinct morphogenetic field and cancer cells permissive to spread and proliferate within that tissue would support the theory (44, 45).

Clinically, the construction of ontogenetic trees guided by the morphology of human embryonic and fetal development and their translation into ontogenetic anatomical maps could be accomplished for any tissue from which cancers originate. Ontogenetic tumor staging and cancer field resections as successfully established for the treatment of colorectal, cervical, and vulvar cancer (12, 40, 41) could be tested for superiority compared to conventional treatment with many other malignant tumor entities. Ontogenetic cancer field-directed treatments are expected to improve the oncologic outcome and decrease treatment-related morbidity particularly for cancers that are diagnosed at a broad spectrum of malignant progression.

## Author Contributions

MH developed the theory of cancer progression by inverse morphogenesis. MH and UB devised the scenario of epigenomic transitions from the scope of self-organizing complex systems. Both authors wrote the manuscript.

### Conflict of Interest Statement

The authors declare that the research was conducted in the absence of any commercial or financial relationships that could be construed as a potential conflict of interest.

## Glossary

**Topobiological information**

Proposed by Gerald Edelman topobiological information is presented to cells by their microenvironment of abutting cells and extracellular matrix as place-dependent molecular signals (epitopes or gradients of soluble substances) or by mechano-transduction of pressure, tension or bioelectrical events.

**Bauplan (Body plan)**

A genetic program unique for a cell type at a developmental stage enabling the cell to perceive topobiological information provided by a morphogenetic field and to respond with cellular activities such as proliferation, migration, aggregation, functional differentiation, apoptosis.

**Morphogenetic field**

A topographically defined territory in the developing organism presenting distinct topobiological information through substance gradients, extracellular matrix, and cell surface epitopes as well as mechanically or electrically.

**Morphogenetic metafield**

An extended topobiological interactive territory including the morphogenetic field of the cell type of interest and the morphogenetic fields of all abutting cell populations that can interchange their bauplans with the bauplan of the cell type of interest due to their epigenomic plasticity.

**Morphogenetic step**

The process of interaction of a cell population with its morphogenetic (meta-)field directed by a developmental stage-associated bauplan and resulting in the emergence of the corresponding tissue's form and function.

**Genomic folding**

A global change of a cell's chromatin conformation with profound consequences for the cell's genetic regulatory network. “Forward” genomic folding denotes epigenetic transitions that increase complexity at decreased plasticity. Conversely, “backward” genomic folding is assumed to represent an inverse process of epigenetic transitions decreasing complexity at increasing plasticity.

**Ontogenetic tree**

Graphic representation of the sequential steps of the developmental path related to a distinct precursor cell population.

**Ontogenetic anatomical map**

Topographical representation of an anatomical region highlighting the mature tissue derivatives of sequential morphogenetic steps by color-coding.

**(Ontogenetic) cancer field**

Spatial distribution of the mature tissue derivatives that emerged from the morphogenetic (meta-)field of a distinct morphogenetic step indicating the territory for local tumor spread.

**Ontogenetic tumor staging**

A classification system for malignant tumors relating the tissues of proven local tumor spread macroscopically (clinically) or microscopically (histopathologically) to the cancer field defined by the morphogenetic step in the development of the tissue from which the malignant tumor has originated. Ontogenetic tumor stages oT1–oT4 are clinically significant in guiding the surgical or radiotherapeutic treatment for local tumor control.

**(Ontogenetic) cancer field resection**

Surgical treatment of malignant tumors for local control respecting the ontogenetic cancer field of the tumor according to its assumed ontogenetic stage. Resection is performed with a wide circumferential tumor-free tissue margin only within the cancer field. At the intact border of the cancer field narrow tumor resection preserving functionally important tissue is appropriate.
