# Supplementary material for: The Order of Cancer: A Theory of Malignant Progression by Inverse Morphogenesis
Source: Front Oncol. 2019 May 29;9:416. doi: 10.3389/fonc.2019.00416 (PMC6548852; doi:10.3389/fonc.2019.00416)
Supplement: Supplementary file 1 [file Data_Sheet_1.PDF]

Supplement

Developmental path of the uterine cervix from the phylotypic stage to maturity

The progenitor tissue for both the genital and urinary tracts appears at the phylotypic stage (Carnegie 11) as bilateral *nephrogenic cords* which are unsegmented solid rods of intermediate mesoderm running caudally lateral to the segmented paraxial mesoderm from the eighth somite. It abuts and interacts with splanchnic coelom medially, somatic coelom and intermediate band ectoderm laterally orchestrated by a common morphogenetic metafield that also involves vascular plexus of the dorsal aorta and of the cardinal veins. Caudally, the intermediate mesoderm cannot be morphologically discriminated from the lateral mesoderm and the anterior cloacal mesenchyme derived from the disc-stalk transitional zone. However, because the distal mesonephric ducts are formed here as well and connect to the primordial hindgut endoderm near the cloacal membrane, these progenitor tissues must also interact with the common morphogenetic metafield. Within this morphogenetic metafield provided by the abdominopelvic somatic and splanchnic coelom, anterior cloacal mesenchyme, primordial hindgut endoderm, cloacal membrane endoderm and intermediate band ectoderm, the intermediate mesoderm differentiates into the *mesonephric system* with tubules and glomeruli and the collecting mesonephric duct and temporarily acts as an excretory organ. Proliferation-induced segregation of the mesonephric system into the primordial genital tract and the metanephric system manifests itself by different morphologic features within regions abutting to the splanchnic coelom as compared to those adjacent to the somatic coelom and the ectoderm. The genital tract primordium now shares the common morphogenetic metafield with the dorsal peritoneal coelom (progeny of the splanchnic coelom) cranially and the splanchnic anterior cloacal mesenchyme (progeny of the anterior cloacal mesenchyme) and the urethrovesical canal endoderm (progeny of the hind gut endoderm) caudally. It gives rise to the *genital ridges* attracting the germ cells from the umbilical vesicle and shifts the mesonephric ducts medially to the lower urethrovesical canal. Interaction with neurovascular primordia are neglected here as they are not essential for understanding the further developmental path.

The following bifurcation, the final one during the embryonic period, separates the primordial genital tract into the gonadal and genital ducts domains enveloped by the genital coelom (progeny of the dorsal splanchnic coelom). The genital ducts domain also abuts and interacts subperitoneally with the central part of the splanchnic anterior cloacal mesenchyme and the urogenital sinus endoderm (progeny of the urethrovesical canal endoderm). This morphogenetic metafield induces the paramesonephric ducts and forms the definitive *genital ducts compartment* by Carnegie stage 23.

The further development of the genital ducts system occurs exclusively within its own morphogenetic field and is sex-dependent. In the female the mesonephric ducts degenerate and the *paramesonephric ducts* differentiate along the craniocaudal axis into the *Fallopian tubes*, *uterine corpus* and *cervix* and *Müllerian vagina*. This process involves the fusion of the paramesonephric ducts from the junction with the urogenital sinus at the sinusal tubercle cranialward to establish the uterovaginal canal. Temporary occlusion of its caudal part manifests as vaginal plate. A fifth subcompartment envelops the other subcompartments as a common neurovascular *Müllerian adventitia*. Each of the four craniocaudal subcompartments develops mesenchymal and epithelial domains. In the uterine cervix the latter also segregates into squamous and cuboid epithelia.

Developmental path of the vulva from the phylotypic stage to maturity

Progenitor tissue of the external genitalia at the phylotypic stage (Carnegie 11) is the *cloacal membrane endoderm* which forms a morphogenetic metafield with the abutting tissues: intermediate band ectoderm, primary hindgut endoderm, anterior cloacal mesenchyme

(derived from the stalk-disc transitional zone), nephrogenic cords, caudal lateral mesenchyme (derived from the caudal eminence) and postcloacal gut. Proliferation-induced segregation of the cloacal membrane endoderm separates the ventral urogenital membrane population from the dorsal anal population. The other tissues of the morphogenetic metafield undergo bifurcational state transitions as well.

During the following developmental stage the *urogenital membrane endoderm* forms a morphogenetic metafield with the abutting next generation tissues: intermembral ectodermal ring, somatic anterior cloacal mesenchyme, and urethrovesical canal endoderm. Urogenital membrane endoderm and the adjacent ectoderm now form the urogenital plate of morphologically uniform cells. As final bifurcation during the embryonic development the *urogenital plate* segregates into a peripheral and a central progeny. The central population degenerates creating the urogenital orifice. The peripheral urogenital plate tissue develops the *external genital compartment* within the morphogenetic metafield that includes, in addition to the peripheral urogenital plate, the abutting next generation tissues: peri-mammary ectoderm, central somatic anterior cloacal mesenchyme and urogenital sinus endoderm. The three concentric substructures of the external genital: peripheral shaft, intermediate glans-labia and central vestibulum can be morphologically discriminated now.

The further development during the fetal period is sex-dependent resulting in the formation of the *vulva* with its *three subcompartments* (peripheral, intermediate and central) each composed of characteristic mesenchyme and epithelium. The epithelium of the intermediate glans-labia subcompartment finally segregates into keratinized and non-keratinized types.
